# *N*,*N*′-(Ethane-1,2-di­yl)bis­(4-chloro­benzene­sulfonamide)

**DOI:** 10.1107/S1600536811028443

**Published:** 2011-08-02

**Authors:** Mohammad T. M. Al-Dajani, Habibah A. Wahab, Shaharum Shamsuddin, Madhukar Hemamalini, Hoong-Kun Fun

**Affiliations:** a School of Pharmaceutical Sciences, Universiti Sains Malaysia, 11800 USM, Penang, Malaysia; b X-ray Crystallography Unit, School of Physics, Universiti Sains Malaysia, 11800 USM, Penang, Malaysia

## Abstract

The title mol­ecule, C_14_H_14_Cl_2_N_2_O_4_S_2_, lies on an inversion center. The mol­ecule is twisted in the region of the sulfonamide group with a C—S—N—C torsion angle of −67.49 (16)°. In the crystal, mol­ecules are connected *via* inter­molecular N—H⋯O and weak C—H⋯O hydrogen bonds, forming layers parallel to (100).

## Related literature

For details of the chemistry of sulfonamides, see: Gowda *et al.* (2003 ▶, 2007 ▶). For related structures, see: O’Connor & Maslen (1965 ▶); Kumar *et al.* (1992 ▶); Shakuntala *et al.* (2011 ▶).
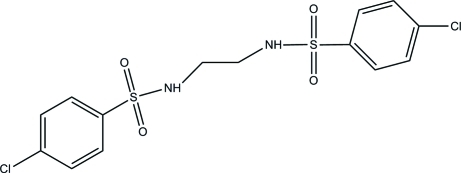


## Experimental

### 

#### Crystal data

C_14_H_14_Cl_2_N_2_O_4_S_2_
*M* *_r_* = 409.29
Monoclinic, 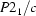

*a* = 13.2640 (5) Å
*b* = 5.3390 (2) Å
*c* = 13.1792 (5) Å
β = 110.270 (1)°
*V* = 875.51 (6) Å^3^
*Z* = 2
Mo *K*α radiation
μ = 0.63 mm^−1^
*T* = 296 K
0.86 × 0.42 × 0.13 mm

#### Data collection

Bruker APEXII DUO CCD area-detector diffractometer
Absorption correction: multi-scan (*SADABS*; Bruker, 2009 ▶) *T*_min_ = 0.613, *T*_max_ = 0.925
25142 measured reflections
3689 independent reflections
2869 reflections with *I* > 2σ(*I*)
*R*_int_ = 0.032

#### Refinement

*R*[*F* ^2^ > 2σ(*F* ^2^)] = 0.054
*wR*(*F* ^2^) = 0.175
*S* = 1.03
3689 reflections
114 parameters
H atoms treated by a mixture of independent and constrained refinement
Δρ_max_ = 0.84 e Å^−3^
Δρ_min_ = −0.71 e Å^−3^

### 

Data collection: *APEX2* (Bruker, 2009 ▶); cell refinement: *SAINT* (Bruker, 2009 ▶); data reduction: *SAINT*; program(s) used to solve structure: *SHELXTL* (Sheldrick, 2008 ▶); program(s) used to refine structure: *SHELXTL*; molecular graphics: *SHELXTL*; software used to prepare material for publication: *SHELXTL* and *PLATON* (Spek, 2009 ▶).

## Supplementary Material

Crystal structure: contains datablock(s) global, I. DOI: 10.1107/S1600536811028443/lh5285sup1.cif

Structure factors: contains datablock(s) I. DOI: 10.1107/S1600536811028443/lh5285Isup2.hkl

Supplementary material file. DOI: 10.1107/S1600536811028443/lh5285Isup3.cml

Additional supplementary materials:  crystallographic information; 3D view; checkCIF report

## Figures and Tables

**Table 1:** Hydrogen-bond geometry (Å, °)

*D*—H⋯*A*	*D*—H	H⋯*A*	*D*⋯*A*	*D*—H⋯*A*
N1—H1*N*1⋯O2^i^	0.79 (3)	2.13 (3)	2.903 (2)	167 (3)
C4—H4*A*⋯O1^ii^	0.93	2.50	3.157 (2)	127

Symmetry codes: (i) 
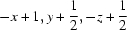
; (ii) 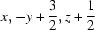.

**Fig. 1.**
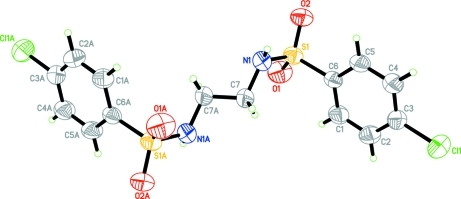
The molecular structure of the title compound showing 50% probability displacement ellipsoids (symmetry code: (A) -x+1, -y+2, -z).

**Fig. 2.**
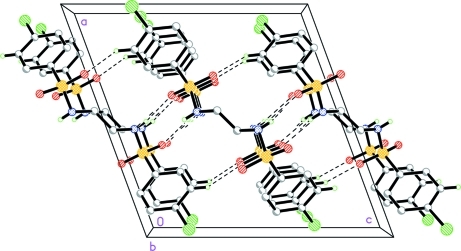
The crystal packing of (I) with dashed lines representing hydrogen bonds.

## supplementary crystallographic information

### Comment

The chemistry of sulfonamides is of interest as they show distinct physical, chemical and biological properties. Many arylsulfonamides and their *N*-halo compounds exhibit pharmacological, fungicidal and herbicidal activities due to their oxidizing action in aqueous, partial aqueous and non-aqueous media. 2-chlorobenzenesulfonamide has been used to explore the substituent effects on the solid state structures of sulfonamides and *N*-haloarylsulfonamides (Gowda *et al.*, 2003, 2007).  The crystal structures of 4-aminobenzenesulfonamide (O'Connor & Maslen, 1965) and 4-methylbenzenesulfonamide (Kumar *et al.*, 1992) have been reported in the literature. In this paper, we present the X-ray single-crystal structure of *N,N'*-(ethane-1,2-diyl)bis(4-chlorobenzenesulfonamide) (I).

The asymmetric unit of the title compound, (I), consists of a half molecule of *N,N'*-(ethane-1,2-diyl)bis(4-chlorobenzenesulfonamide). The other half is generated by a crystallographic inversion center, as shown in Fig. 1. The molecule is twisted at the S atom with an C-S-N-C torsion angle of -67.49 (16)°. This value agrees with that previously reported for the crystal structure of 4-Chloro-*N*-(2,6-dimethylphenyl)benzene sulfonamide (Shakuntala *et al.*, 2011). In the crystal, molecules are connected *via* N1—H1N1···O2^ii^ and C4—H4A···O1^iii^ (Table 1) hydrogen bonds (Fig. 2) forming layers parallel to (100).

### Experimental

In a round bottom flask, 25ml of toluene was mixed with 4-chlorobenzenesulfonyl chloride (0.02 mol, 3.5 g) with stirring.  Drops of ethylenediamine (0.01mol, 0.5 g ) were added and the mixture was refluxed for 30 min. The yellow gum formed was dissolved in hot water and sodium bicarbonate was added. The yellow precipitate formed was dissolved in methanol at 333K, yielding colourless crystals.

### Refinement

Atom H1N1 was located from a difference Fourier maps and refined freely [N–H = 0.78 (3) Å]. The remaining H atoms were positioned geometrically [C–H = 0.93–0.97 Å] and were refined using a riding model, with *U*_iso_(H) = 1.2 *U*_eq_(C).

### Crystal data

**Table:**

C_14_H_14_Cl_2_N_2_O_4_S_2_	*F*(000) = 420
*M**_r_* = 409.29	*D*_x_ = 1.553 Mg m^−^^3^
Monoclinic, *P*2_1_/*c*	Mo *K*α radiation, λ = 0.71073 Å
Hall symbol: -P 2ybc	Cell parameters from 9471 reflections
*a* = 13.2640 (5) Å	θ = 3.3–33.5°
*b* = 5.3390 (2) Å	µ = 0.63 mm^−^^1^
*c* = 13.1792 (5) Å	*T* = 296 K
β = 110.270 (1)°	Plate, colourless
*V* = 875.51 (6) Å^3^	0.86 × 0.42 × 0.13 mm
*Z* = 2	

### Data collection

**Table:**

Bruker APEXII DUO CCD area-detector diffractometer	3689 independent reflections
Radiation source: fine-focus sealed tube	2869 reflections with *I* > 2σ(*I*)
graphite	*R*_int_ = 0.032
φ and ω scans	θ_max_ = 34.5°, θ_min_ = 3.1°
Absorption correction: multi-scan (*SADABS*; Bruker, 2009)	*h* = −21→21
*T*_min_ = 0.613, *T*_max_ = 0.925	*k* = −8→8
25142 measured reflections	*l* = −19→20

### Refinement

**Table:**

Refinement on *F*^2^	Secondary atom site location: difference Fourier map
Least-squares matrix: full	Hydrogen site location: inferred from neighbouring sites
*R*[*F*^2^ > 2σ(*F*^2^)] = 0.054	H atoms treated by a mixture of independent and constrained refinement
*wR*(*F*^2^) = 0.175	*w* = 1/[σ^2^(*F*_o_^2^) + (0.0979*P*)^2^ + 0.221*P*] where *P* = (*F*_o_^2^ + 2*F*_c_^2^)/3
*S* = 1.03	(Δ/σ)_max_ < 0.001
3689 reflections	Δρ_max_ = 0.84 e Å^−^^3^
114 parameters	Δρ_min_ = −0.71 e Å^−^^3^
0 restraints	Extinction correction: *SHELXTL* (Sheldrick, 2008), Fc^*^=kFc[1+0.001xFc^2^λ^3^/sin(2θ)]^-1/4^
Primary atom site location: structure-invariant direct methods	Extinction coefficient: 0.037 (6)

### Special details

**Table:**

Geometry. All s.u.'s (except the s.u. in the dihedral angle between two l.s. planes) are estimated using the full covariance matrix. The cell s.u.'s are taken into account individually in the estimation of s.u.'s in distances, angles and torsion angles; correlations between s.u.'s in cell parameters are only used when they are defined by crystal symmetry. An approximate (isotropic) treatment of cell s.u.'s is used for estimating s.u.'s involving l.s. planes.
Refinement. Refinement of F^2^ against ALL reflections. The weighted R-factor wR and goodness of fit S are based on F^2^, conventional R-factors R are based on F, with F set to zero for negative F^2^. The threshold expression of F^2^ > 2σ(F^2^) is used only for calculating R-factors(gt) etc. and is not relevant to the choice of reflections for refinement. R-factors based on F^2^ are statistically about twice as large as those based on F, and R- factors based on ALL data will be even larger.

### Fractional atomic coordinates and isotropic or equivalent isotropic displacement parameters (Å^2^)

**Table:**

	*x*	*y*	*z*	*U*_iso_*/*U*_eq_	
Cl1	0.05348 (6)	1.46054 (14)	0.19657 (6)	0.0881 (2)	
S1	0.35715 (3)	0.67863 (7)	0.09928 (3)	0.04701 (14)	
O1	0.30882 (14)	0.5925 (3)	−0.00946 (11)	0.0722 (4)	
O2	0.39055 (12)	0.5017 (2)	0.18611 (12)	0.0615 (3)	
N1	0.46306 (13)	0.8326 (3)	0.10557 (14)	0.0537 (3)	
C1	0.18737 (14)	1.0017 (4)	0.04025 (13)	0.0572 (4)	
H1A	0.1784	0.9592	−0.0308	0.069*	
C2	0.12110 (16)	1.1788 (4)	0.06200 (16)	0.0648 (5)	
H2A	0.0680	1.2581	0.0056	0.078*	
C3	0.13465 (14)	1.2362 (4)	0.16811 (17)	0.0564 (4)	
C4	0.21201 (17)	1.1220 (4)	0.25315 (15)	0.0602 (4)	
H4A	0.2191	1.1612	0.3240	0.072*	
C5	0.27916 (15)	0.9482 (4)	0.23194 (12)	0.0543 (4)	
H5A	0.3325	0.8707	0.2888	0.065*	
C6	0.26714 (11)	0.8888 (3)	0.12547 (11)	0.0428 (3)	
C7	0.45903 (17)	1.0240 (4)	0.02466 (18)	0.0635 (5)	
H7A	0.4710	1.1877	0.0587	0.076*	
H7B	0.3884	1.0241	−0.0311	0.076*	
H1N1	0.496 (2)	0.868 (6)	0.166 (2)	0.076 (8)*	

### Atomic displacement parameters (Å^2^)

**Table:**

	*U*^11^	*U*^22^	*U*^33^	*U*^12^	*U*^13^	*U*^23^
Cl1	0.0889 (4)	0.0823 (4)	0.1081 (5)	0.0331 (3)	0.0532 (4)	0.0217 (3)
S1	0.0555 (2)	0.0437 (2)	0.03914 (19)	−0.00060 (14)	0.01292 (15)	−0.00090 (12)
O1	0.0873 (10)	0.0712 (9)	0.0468 (6)	0.0035 (8)	0.0090 (6)	−0.0168 (6)
O2	0.0717 (8)	0.0495 (7)	0.0620 (7)	0.0049 (6)	0.0214 (6)	0.0146 (5)
N1	0.0538 (7)	0.0590 (8)	0.0516 (7)	0.0028 (6)	0.0226 (6)	0.0065 (6)
C1	0.0508 (8)	0.0756 (12)	0.0390 (7)	0.0038 (7)	0.0079 (6)	0.0079 (7)
C2	0.0524 (9)	0.0799 (13)	0.0563 (9)	0.0152 (8)	0.0113 (7)	0.0193 (9)
C3	0.0522 (8)	0.0566 (9)	0.0653 (10)	0.0068 (7)	0.0265 (7)	0.0122 (8)
C4	0.0711 (11)	0.0637 (10)	0.0484 (8)	0.0112 (9)	0.0241 (8)	0.0060 (7)
C5	0.0620 (9)	0.0605 (9)	0.0374 (6)	0.0110 (7)	0.0135 (6)	0.0078 (6)
C6	0.0431 (6)	0.0460 (7)	0.0366 (5)	−0.0031 (5)	0.0104 (5)	0.0047 (5)
C7	0.0724 (11)	0.0577 (10)	0.0775 (12)	0.0207 (8)	0.0477 (10)	0.0202 (9)

### Geometric parameters (Å, °)

**Table:**

Cl1—C3	1.7359 (19)	C2—C3	1.381 (3)
S1—O1	1.4282 (14)	C2—H2A	0.9300
S1—O2	1.4308 (13)	C3—C4	1.373 (3)
S1—N1	1.6048 (16)	C4—C5	1.380 (3)
S1—C6	1.7574 (16)	C4—H4A	0.9300
N1—C7	1.465 (2)	C5—C6	1.393 (2)
N1—H1N1	0.78 (3)	C5—H5A	0.9300
C1—C6	1.386 (2)	C7—C7^i^	1.469 (3)
C1—C2	1.387 (3)	C7—H7A	0.9700
C1—H1A	0.9300	C7—H7B	0.9700
			
O1—S1—O2	119.79 (10)	C2—C3—Cl1	119.94 (14)
O1—S1—N1	107.30 (10)	C3—C4—C5	119.05 (17)
O2—S1—N1	105.90 (9)	C3—C4—H4A	120.5
O1—S1—C6	107.66 (8)	C5—C4—H4A	120.5
O2—S1—C6	107.92 (8)	C4—C5—C6	120.03 (15)
N1—S1—C6	107.76 (8)	C4—C5—H5A	120.0
C7—N1—S1	120.62 (14)	C6—C5—H5A	120.0
C7—N1—H1N1	116 (2)	C1—C6—C5	120.40 (16)
S1—N1—H1N1	110 (2)	C1—C6—S1	119.90 (12)
C6—C1—C2	119.32 (16)	C5—C6—S1	119.66 (11)
C6—C1—H1A	120.3	N1—C7—C7^i^	110.75 (19)
C2—C1—H1A	120.3	N1—C7—H7A	109.5
C3—C2—C1	119.42 (16)	C7^i^—C7—H7A	109.5
C3—C2—H2A	120.3	N1—C7—H7B	109.5
C1—C2—H2A	120.3	C7^i^—C7—H7B	109.5
C4—C3—C2	121.75 (18)	H7A—C7—H7B	108.1
C4—C3—Cl1	118.31 (16)		
			
O1—S1—N1—C7	48.19 (17)	C2—C1—C6—S1	−176.04 (15)
O2—S1—N1—C7	177.22 (15)	C4—C5—C6—C1	−0.5 (3)
C6—S1—N1—C7	−67.49 (16)	C4—C5—C6—S1	176.93 (15)
C6—C1—C2—C3	−1.0 (3)	O1—S1—C6—C1	−20.18 (17)
C1—C2—C3—C4	−0.2 (3)	O2—S1—C6—C1	−150.79 (14)
C1—C2—C3—Cl1	179.13 (16)	N1—S1—C6—C1	95.26 (15)
C2—C3—C4—C5	1.1 (3)	O1—S1—C6—C5	162.35 (16)
Cl1—C3—C4—C5	−178.26 (16)	O2—S1—C6—C5	31.74 (16)
C3—C4—C5—C6	−0.7 (3)	N1—S1—C6—C5	−82.21 (15)
C2—C1—C6—C5	1.4 (3)	S1—N1—C7—C7^i^	−126.2 (2)

Symmetry codes: (i) −*x*+1, −*y*+2, −*z*.

### Hydrogen-bond geometry (Å, °)

**Table:**

*D*—H···*A*	*D*—H	H···*A*	*D*···*A*	*D*—H···*A*
N1—H1N1···O2^ii^	0.79 (3)	2.13 (3)	2.903 (2)	167 (3)
C4—H4A···O1^iii^	0.93	2.50	3.157 (2)	127

Symmetry codes: (ii) −*x*+1, *y*+1/2, −*z*+1/2; (iii) *x*, −*y*+3/2, *z*+1/2.

## References

[bb1] Bruker (2009). *APEX2*, *SAINT* and *SADABS* Bruker AXS Inc., Madison, Wisconsin, USA.

[bb2] Gowda, B. T., Babitha, K. S., Svoboda, I. & Fuess, H. (2007). *Acta Cryst.* E**63**, o3245. 10.1107/S1600536807062137 PMC2915044 21200965

[bb3] Gowda, B. T., Jyothi, K., Kozisek, J. & Fuess, H. (2003). *Z. Naturforsch. Teil A*, **58**, 656–660.

[bb4] Kumar, S. V., Senadhi, S. E. & Rao, L. M. (1992). *Z. Kristallogr.* **202**, 1–6.

[bb5] O’Connor, B. H. & Maslen, E. N. (1965). *Acta Cryst.* **18**, 363–366.

[bb6] Shakuntala, K., Foro, S. & Gowda, B. T. (2011). *Acta Cryst.* E**67**, o1401. 10.1107/S160053681101717X PMC3120512 21754786

[bb7] Sheldrick, G. M. (2008). *Acta Cryst.* A**64**, 112–122. 10.1107/S0108767307043930 18156677

[bb8] Spek, A. L. (2009). *Acta Cryst.* D**65**, 148–155. 10.1107/S090744490804362X PMC2631630 19171970

